# From guidelines to clinical practice: the impact of sentinel lymph node mapping on surgical management in endometrial cancer

**DOI:** 10.25122/jml-2025-0089

**Published:** 2025-06

**Authors:** Alexandru Blidaru, Maria-Bianca Anca-Stanciu, Andrei Manu, Bogdan Cătălin Coroleucă, Ciprian Andrei Coroleucă, Elvira Brătilă

**Affiliations:** 1 Department of Surgical Oncology, Carol Davila University of Medicine and Pharmacy, Bucharest, Romania; 2 Institute of Oncology 'Prof. Dr. Alexandru Trestioreanu', Bucharest, Romania; 3 Doctoral School, Carol Davila University of Medicine and Pharmacy, Bucharest, Romania; 4 Prof. Dr. Panait Sârbu Clinical Hospital of Obstetrics and Gynecology, Carol Davila University of Medicine and Pharmacy, Bucharest, Romania

**Keywords:** endometrial cancer, sentinel lymph node, indocyanine green, laparoscopic surgery, ultrastaging

## Abstract

Sentinel lymph node (SLN) mapping using indocyanine green (ICG) fluorescence has emerged as a less invasive alternative to systematic lymphadenectomy in the surgical management of early-stage endometrial cancer. This study aimed to evaluate the feasibility, accuracy, and clinical outcomes of SLN mapping integrated into laparoscopic staging for endometrial cancer based on our institutional experience. A retrospective study was conducted on 29 patients with early-stage endometrial cancer who underwent laparoscopic hysterectomy with bilateral salpingo-oophorectomy and SLN mapping using ICG. Detection rates, histopathological findings, complication rates, and follow-up outcomes were recorded. SLN detection was successful in 100% of patients, with bilateral mapping achieved in 75.9% of cases. Metastatic involvement was found in 13.8% of cases, with micrometastases detected through ultrastaging. No significant intraoperative or postoperative complications were reported. Risk-adapted adjuvant treatment was administered according to ESGO/ESTRO/ESP guidelines. After a median follow-up of 18 months, 93.1% of patients remained disease-free. SLN mapping with ICG is a reliable and safe technique for lymphatic staging in endometrial cancer, enabling accurate nodal assessment while minimizing surgical morbidity. These findings support the routine implementation of this approach in the laparoscopic management of early-stage disease.

## INTRODUCTION

Endometrial cancer (EC) is increasingly prevalent and currently represents one of the most common gynecologic malignancies worldwide. In the United States alone, uterine cancer ranks second among cancers in women, with an estimated 66,570 new cases and 12,940 deaths reported for 2021 [1]. Similarly, in Europe, there were approximately 121,578 new cases and 29,638 deaths reported in 2018, reflecting an upward trend associated with an aging population and rising obesity rates [2]. The primary symptom leading to an early diagnosis of EC is postmenopausal bleeding, resulting in generally favorable outcomes and high survival rates when detected at an early stage. However, advanced-stage or recurrent disease typically has a significantly worse prognosis [3,4]. Survival rates also vary by histological subtype, with endometrioid carcinoma presenting a considerably better prognosis compared to clear-cell or serous subtypes, achieving 5-year survival rates of approximately 85% for FIGO stage I and 75% for stage II [5].

Surgery remains the cornerstone of treatment for endometrial cancer, primarily involving hysterectomy with bilateral salpingo-oophorectomy. Nevertheless, the role of lymphadenectomy remains controversial, given the significant risks associated with the procedure, such as increased morbidity and postoperative complications [6-8]. To mitigate these risks, sentinel lymph node (SLN) mapping has emerged as a minimally invasive alternative, effectively reducing morbidity without compromising diagnostic accuracy [9]. SLN is defined as the first node that receives lymphatic drainage from the primary tumor, thereby accurately reflecting the metastatic status of the regional lymphatic basin [10].

Minimally invasive surgery, particularly laparoscopic approaches, has become the standard of care in the surgical management of early-stage endometrial cancer due to its favorable perioperative profile and comparable oncologic outcomes to open surgery [11]. The introduction of indocyanine green (ICG) fluorescence has further enhanced the precision of SLN mapping, allowing for clear visualization of lymphatic pathways with minimal invasiveness [12]. Furthermore, the adoption of ESGO/ESTRO/ESP risk stratification enables clinicians to individualize adjuvant treatment, balancing oncologic safety with the need to avoid overtreatment [13].

According to the latest guidelines from the European Society of Gynecological Oncology (ESGO) and the National Comprehensive Cancer Network (NCCN), SLN mapping is recommended as an acceptable alternative to comprehensive lymphadenectomy in patients with early-stage endometrial cancer, particularly in clinically node-negative cases. ESGO emphasizes the use of ICG fluorescent dye as the preferred method due to its high sensitivity and accuracy compared to traditional methods such as blue dye and radiocolloid [14]. Similarly, NCCN guidelines endorse SLN mapping with ultrastaging, noting its utility in enhancing diagnostic accuracy and staging precision, thus potentially influencing adjuvant treatment decisions [15]. Both guidelines, as shown in Table 1, emphasize the importance of surgeons adhering strictly to established protocols and algorithms to optimize detection rates and minimize false-negative outcomes [14,15].

**Table 1:** Comparative summary of Sentinel Lymph Node Mapping recommendations from major international guidelines

Criteria	ESGO (2021)	NCCN (2024)
Preferred dye/method	Indocyanine green (ICG)	Indocyanine green (ICG)
Clinical stage indicated	Early-stage (I-II), clinically node-negative	Early-stage (I-II), clinically node-negative
Ultrastaging recommended	Yes	Yes
Algorithm adherence required	Yes	Yes
Role in clinical decision-making	Influences adjuvant therapy	Influences adjuvant therapy
Alternative to lymphadenectomy	Acceptable alternative	Acceptable alternative

ESGO, European Society of Gynaecological Oncology; NCCN, National Comprehensive Cancer Network; ICG, Indocyanine Green.

In our institution, we adopted the SLN mapping technique using ICG fluorescence, integrating it into routine surgical staging for early-stage endometrial cancer. This article aims to present our institutional experience, analyzing our results regarding detection rates, accuracy, and associated clinical outcomes. We will discuss our results in the context of current guidelines and literature evidence, evaluating the impact of adopting SLN mapping on surgical morbidity and patient prognosis.

## MATERIAL AND METHODS

A retrospective observational study was conducted, including 29 patients diagnosed with early-stage endometrial cancer who underwent laparoscopic surgical treatment with sentinel lymph node mapping using ICG at the Clinical Hospital of Obstetrics and Gynecology “Prof. Dr. Panait Sarbu” during 2023-2024. Inclusion criteria were patients diagnosed with clinically early-stage (FIGO stage I-II), histologically confirmed endometrial carcinoma, suitable for laparoscopic surgery, and without clinical or imaging suspicion of nodal or distant metastases. Exclusion criteria included advanced-stage endometrial cancer (FIGO stage III-IV), contraindications for laparoscopic procedures, known allergies to indocyanine green dye, pregnancy, or refusal of informed consent. All patients underwent standardized surgical staging procedures, which included laparoscopic hysterectomy, bilateral salpingo-oophorectomy, and sentinel lymph node mapping using the ICG fluorescence technique. Patients were systematically followed up clinically, and data regarding demographic characteristics, surgical outcomes, SLN detection rates, histopathological findings, postoperative complications, and follow-up results were collected and analyzed.

## RESULTS

**Figure 1:**
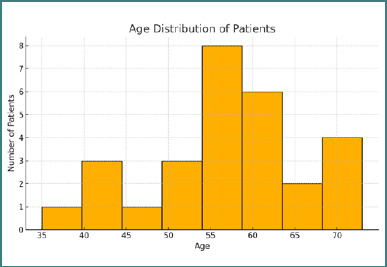
Histogram representing the age distribution of patients included in the study

A total of 29 patients diagnosed with early-stage endometrial cancer underwent total laparoscopic hysterectomy with bilateral salpingo-oophorectomy and sentinel lymph node mapping using ICG fluorescence. All procedures were performed via a minimally invasive approach, following a standardized protocol for SLN identification. The mean age of the patients was 57.6 years, with a median age of 57.5 years (range: 35–73 years). The age distribution is shown in Figure 1. The average body mass index (BMI) of the patients was 33.9 kg/m^2^, with a median value of 33.5 kg/m^2^ (range: 20.01–51.6 kg/m^2^), consistent with the association between endometrial cancer and obesity. Among the 29 patients, 10 (34.5%) had a known diagnosis of arterial hypertension, and three (10.3%) were diagnosed with type 2 diabetes mellitus. Regarding lifestyle-related risk factors, seven patients (24.1%) were active smokers at the time of diagnosis.

**Figure 2:**
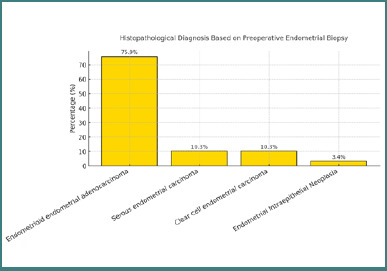
Histopathological diagnosis based on preoperative endometrial biopsy

Preoperative endometrial biopsy revealed that the majority of patients (22 out of 29; 75.9%) were diagnosed with endometrioid endometrial adenocarcinoma. Serous endometrial carcinoma and clear cell carcinoma were each identified in three patients (10.3%), while one patient (3.4%) was diagnosed with endometrial intraepithelial neoplasia (Figure 2).

**Figure 3:**
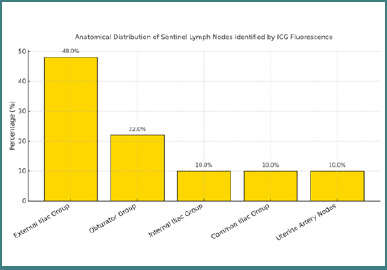
Percentage distribution of sentinel lymph node locations identified by indocyanine green fluorescence during laparoscopic staging for endometrial cancer

During laparoscopic procedures, sentinel lymph nodes were identified using indocyanine green fluorescence. A total of 50 sentinel lymph nodes were detected. Their anatomical distribution was as follows: 24 nodes (48%) in the external iliac group, 11 nodes (22%) in the obturator group, five nodes (10%) in the internal iliac group, five nodes (10%) in the common iliac group, and five nodes (10%) in the region adjacent to the uterine artery. These data are illustrated in Figure 3. SLN detection was successful in all 29 patients, yielding a detection rate of 100%. Bilateral SLN mapping was achieved in 22 cases (75.9%), while in seven patients (24.1%), the sentinel nodes were detected unilaterally. These results confirm the feasibility and reproducibility of SLN identification using indocyanine green fluorescence during laparoscopic staging for endometrial cancer.

Histopathological evaluation of the sentinel lymph nodes, including ultrastaging, revealed lymph node metastases in 4 out of the 29 patients (13.8%). These cases were considered sentinel lymph node-positive, indicating tumor infiltration at the nodal level. All positive nodes were detected through the indocyanine green–guided mapping technique and confirmed by microscopic examination, including serial sectioning and immunohistochemistry when required. Among the 29 patients, SLN metastases were identified in four cases (13.8%) through ultrastaging. Of these, three patients presented with micrometastases, and one had macrometastatic involvement. All SLN-positive cases were associated with endometrioid histology grade 2 or 3 and demonstrated the presence of lymphovascular space invasion (LVSI).

**Figure 4:**
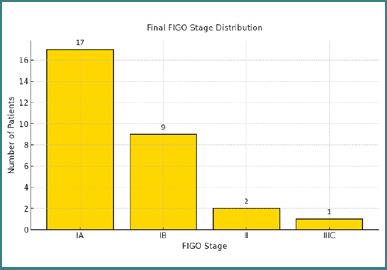
Distribution of final FIGO stages in the study population

The final histopathological analysis confirmed the preoperative diagnosis in all cases. Based on the surgical specimens, tumor grading revealed that 13 patients (44.8%) had G2 tumors, eight (27.6%) had G1 tumors, six (20.7%) had G3 tumors, and two (6.9%) were classified as G1–G2. Regarding staging, 17 patients (58.6%) were diagnosed with FIGO stage IA, nine (31.0%) with stage IB, two (6.9%) with stage II, and one patient (3.4%) was staged as IIIC. These results are illustrated in Figure 4.

Immunohistochemical analysis was performed in 11 of the 29 patients (Table 2). Estrogen receptor expression was positive in 10 patients, and progesterone receptor was positive in nine cases. All cases showed a wild-type p53 staining pattern. Ki67 proliferation index was elevated (>50%) in four cases. HER2 positivity was observed in six patients, while vimentin expression was consistently positive in the evaluated samples. Mismatch repair (MMR) protein analysis showed that seven patients (63.6%) were microsatellite stable (MSS), and four patients (36.4%) exhibited high microsatellite instability (MSI-H).

**Table 2:** Summary of immunohistochemical markers evaluated

Marker	Positive	Negative / Not Reported
**ER**	10	1
**PR**	9	2
**p53 (wild type)**	11	0
**Ki67 >50%**	4	7
**HER2**	6	5
**Vimentin**	7	0
**MSS**	7	4
**MSI-H**	4	7

ER, Estrogen Receptor; PR, Progesterone Receptor; HER2, Human Epidermal Growth Factor Receptor 2; MSS, Microsatellite Stable; MSI-H, Microsatellite Instability-High

No significant short- or long-term postoperative complications were recorded in the study cohort. Intraoperative blood loss was minimal in all patients. No cases of excessive postoperative lymphorrhagia, lymphocele formation, wound infection, nerve injury, or lower limb lymphedema were observed. These complications are typically associated with lymphadenectomy. Sentinel lymph node mapping was seamlessly integrated into the laparoscopic staging procedures without a significant prolongation of operative time. The additional time required for ICG injection and lymphatic mapping did not interfere with the surgical workflow.

All patients were stratified into prognostic risk groups according to ESGO/ESTRO/ESP guidelines. Based on histology, grade, myometrial invasion, and lymphovascular space invasion (LVSI), adjuvant therapy was administered as appropriate for each risk category. Risk groups and corresponding treatments included:

1. *Low risk* – no adjuvant therapy;
2. *Intermediate risk* – vaginal brachytherapy;
3. *High–intermediate risk* – pelvic external beam radiotherapy with or without chemotherapy;
4. *High risk* – combined chemotherapy and radiotherapy.

**Figure 5:**
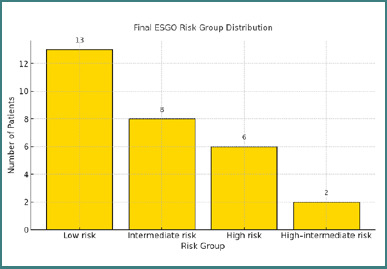
Distribution of patients across ESGO/ESTRO/ESP prognostic risk groups

All patients received adjuvant treatment tailored to their risk profile. Nineteen patients (65.5%) were classified as low risk, most commonly with endometrioid histology, stage IA tumors, grades 1–2, and absent or focal lymphovascular space invasion (LVSI). Five patients (17.2%) were considered intermediate risk, based on either stage IB grade 1–2 tumors or stage IA grade 3 tumors without LVSI. Two patients (6.9%) were categorized as high–intermediate risk, which included cases with substantial LVSI or stage II disease. Additionally, six patients (20.7%) were classified as high-risk due to non-endometrioid histology and/or advanced-stage disease. The distribution of risk groups is illustrated in Figure 5.

After a median follow-up period of 18 months (range: 12–26 months), 27 out of 29 patients (93.1%) remained disease-free. Two patients (6.9%) experienced isolated local recurrences at the vaginal vault, which were detected during routine surveillance. No distant recurrences or disease-related deaths were reported during the follow-up interval.

## DISCUSSION

These findings support the safety and feasibility of sentinel lymph node mapping using indocyanine green in laparoscopic management of early-stage endometrial cancer, with low complication rates, accurate staging, and individualized risk-adapted adjuvant therapy. Our findings support the growing body of evidence that sentinel lymph node mapping with ICG is a reliable method for nodal staging in early-stage endometrial cancer, with a high detection rate and low morbidity.

This study confirms that sentinel lymph node mapping using ICG fluorescence during laparoscopic staging for endometrial cancer is a reliable and safe alternative to systematic lymphadenectomy. In our cohort of 29 patients, SLN detection was achieved in 100% of cases, with a bilateral mapping rate of 75.9%. These results are comparable to those reported in the FIRES trial and other prospective studies, reinforcing the feasibility and reproducibility of this technique in clinical practice [16]. Similarly, the SENTI-ENDO study demonstrated an 88.8% detection rate, a sensitivity of 84%, and a negative predictive value of 97% [17]. In our cohort, we achieved a 100% SLN detection rate and a 75.9% bilateral mapping rate, values comparable or even superior to those reported in the aforementioned studies.

Importantly, ultrastaging contributed significantly to the diagnostic accuracy of nodal evaluation. Among the four patients with metastatic SLNs, three had micrometastases that were identified only through serial sectioning and immunohistochemistry. This emphasizes the importance of applying advanced pathological techniques in SLN evaluation, as standard hematoxylin and eosin (H&E) staining alone may not be sufficient to detect low-volume disease. Furthermore, the identification of metastases in 13.8% of patients, including micrometastases revealed through ultrastaging, underscores the importance of advanced pathological assessment—a point also emphasized in the SENTI-ENDO trial [17]. These comparisons situate our results within the context of established evidence, supporting the adoption of SLN mapping with ICG as a standard component of laparoscopic staging in early-stage endometrial cancer.

Another strength of our study lies in the low complication rate. No cases of excessive blood loss, lymphorrhagia, lymphocele, lymphedema, or wound infection were observed. These findings support the hypothesis that SLN mapping significantly reduces the morbidity associated with full lymphadenectomy without compromising the accuracy of staging. Systematic lymphadenectomy (LAD) in endometrial cancer has been associated with significant postoperative complications [18]. A retrospective study involving 232 patients revealed that those undergoing LAD experienced higher rates of postoperative complications, including lymphoceles and lymphedema, compared to those who did not undergo LAD [18]. Notably, 40.9% of patients who underwent LAD developed long-term lymphatic complications, such as lymphoceles and lymphedema, adversely affecting their quality of life [18]. In contrast, SLN mapping has demonstrated a favorable safety profile. A study comparing SLN mapping alone to SLN mapping combined with LAD found that patients undergoing SLN mapping alone had significantly lower overall complication rates (14.2%) compared to those undergoing both procedures (33.3%) [19]. These findings suggest that SLN mapping not only provides accurate staging information but also minimizes surgical morbidity, making it a preferable approach in the management of early-stage endometrial cancer.

The risk stratification, as outlined in the ESGO/ESTRO/ESP consensus, enabled an individualized approach to adjuvant therapy. Most patients in our cohort were classified as low-risk and received no further treatment, while those in intermediate or high intermediate–risk groups received appropriate adjuvant therapies. The absence of distant recurrence during the median 18-month follow-up suggests that this risk-adapted approach may be both effective and oncologically safe.

Two cases of local recurrence were observed, both located at the vaginal vault, a known site of recurrence in endometrial cancer. These were detected through routine surveillance, and both patients received salvage treatment.

While the results are promising, the study has certain limitations. Firstly, the relatively small sample size (*n* = 29) from a single center restricts the generalizability of our findings. Secondly, the median follow-up period of 18 months is relatively short, limiting definitive conclusions regarding long-term recurrence-free and overall survival outcomes. Lastly, the absence of a control group undergoing systematic lymphadenectomy prevents a direct comparison of staging sensitivity and long-term oncologic outcomes between the two surgical approaches. Future larger, multicenter prospective studies with extended follow-up and comparative groups are needed to validate these preliminary results.

## CONCLUSION

Sentinel lymph node mapping using ICG fluorescence is a reliable and reproducible method for lymphatic staging in patients with early-stage endometrial cancer. Our institutional experience confirms a high detection rate (100%) and a favorable bilateral mapping rate (75.9%), in line with the results of large-scale studies. Ultrastaging significantly improved nodal assessment, allowing the detection of micrometastases in cases that would have otherwise been missed through conventional histopathology. SLN mapping demonstrated an excellent safety profile, with no significant intraoperative or postoperative complications observed, thus avoiding the morbidity associated with systematic lymphadenectomy. The incorporation of ESGO-based risk classification allowed for individualized, risk-adapted adjuvant therapy, which was associated with excellent short-term oncologic outcomes. Based on our results, sentinel lymph node mapping with ICG should be considered a standard component of the laparoscopic surgical approach for early-stage endometrial cancer, contributing to accurate staging, reduced morbidity, and optimized therapeutic decision-making.

## Data Availability

Further data is available from the corresponding author on reasonable request.
